# To Express or to End? Personality Traits Are Associated With the Reasons and Patterns for Using Emojis and Stickers

**DOI:** 10.3389/fpsyg.2020.01076

**Published:** 2020-06-09

**Authors:** Siying Liu, Renji Sun

**Affiliations:** ^1^Institute of Linguistics, Shanghai International Studies University, Shanghai, China; ^2^School of Business, East China University of Political Science and Law, Shanghai, China

**Keywords:** emojis, stickers, personality traits, computer-mediated communication, usage patterns

## Abstract

Emojis and stickers are becoming increasingly popular in computer-mediated communications. The present study examined the associations between personality traits and people’s reasons and patterns for using both emojis and stickers. Participants (*n* = 312) completed three online questionnaires assessing shyness, the Big Five personality traits, and why and how they used emojis and stickers. Results revealed that shyness, neuroticism, extraversion, and agreeableness were correlated with different reasons of usage. Moreover, some participants exhibited a tendency to adjust frequency of usage depending on who the target person was and whether they were in a private or group chat. People who showed such tendencies were found to differ in personality with those who did not. Some differences in usage patterns were also observed between emojis and stickers. Together, the present study has produced more insight into how emojis and stickers can help people with different personality traits to achieve different purposes in their daily communication.

## Introduction

**FIGURE 1:**
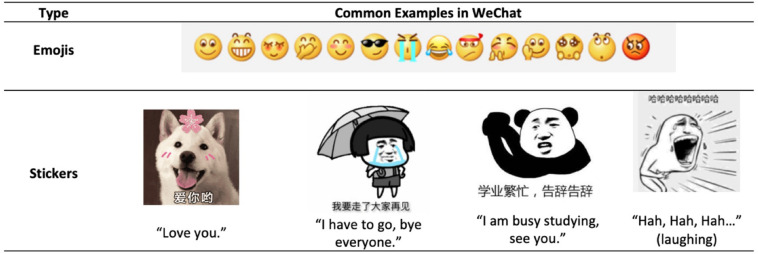
Examples of emojis and stickers that are available in WeChat.

Human communication is always evolving (Crystal, 2006). With the advent of social media and instant messaging services (e.g., Apple iMessage, WhatsApp, and WeChat), computer-mediated communication (CMC) has become an indispensable part in many people’s daily lives (Alshenqeeti, 2016; Riordan, 2017; Prada et al., 2018). While people adopt a variety of emotional cues, such as facial expressions and voice tones, when conversing face to face, they also find the need to do so in CMC (Derks et al., 2008). This has led to the birth of a new form of “language” named emojis (Krohn, 2004; Marengo et al., 2017). Emojis are small symbols available on standardized keyboards that can be inserted within written texts to represent a wide range of faces, objects, and ideas. They originated from emoticons, which were facial expressions formed by punctuation marks (Cramer et al., 2016; Lu et al., 2016; Tang and Hew, 2019). Recently, a new type of emoji called a sticker is also widely adopted in the instant messaging world (Chen and Siu, 2017; Zhou et al., 2017). In comparison to emojis, stickers are animated or static images that are usually bigger and therefore must be sent separately from the written texts (Zhou et al., 2017). They can be combined with short texts to represent more complex ideas, such as environmental descriptions, body language, and textual illustrations (see Figure 1 for examples of emojis and stickers available on the instant messaging platform WeChat) (Lee et al., 2016).

Research evidence suggests that emojis and their related products share many similarities with early pictorial or symbolic languages that convey information through imagery (Dresner and Herring, 2014; Kern, 2015; Alshenqeeti, 2016). From a linguistic point of view, some researchers regard emojis as representations of morphemes and words (Kavanagh, 2016). Others believe emojis are non-verbal cues that serve paralinguistic functions, such as facilitating emotional expression (e.g., showing affection, sarcasm, and euphemisms) and providing greater depth to the content of the message (Walther and D’Addario, 2001; Krohn, 2004; Azuma and Ebner, 2008; Lo, 2008; Azuma, 2012; Jibril and Abdullah, 2013; Alshenqeeti, 2016; Thompson and Filik, 2016; Prada et al., 2018). In either case, emojis are seen as expansions of people’s linguistic ability and offer a more innovative way for people to interact in CMC (Danesi, 2016).

Emojis have attracted great empirical attention, and one major line of research focuses on why people use them. Research that examined different CMC platforms, such as emails, text messages, and social networking sites, have found that one of the most prominent reasons that people use emojis and stickers is to communicate emotions and feelings (Derks et al., 2008; Dresner and Herring, 2010; Luor et al., 2010; Kaye et al., 2017; Hu et al., 2017; Zhou et al., 2017; Prada et al., 2018). For example, Lu et al. (2016) analyzed messages from over 3 million smartphone users across 212 countries and found that the one of the most common reasons people use emojis is to express emotion. In another study by Kaye et al. (2016), participants provided open-ended accounts of their reasons for using emoticons. Results revealed that emoticon usage is mainly driven by a motivation to establish emotional tones and create a positive mood in conversations. These results were further confirmed by Prada et al. (2018) who analyzed self-reports from 474 participants and discovered emotion expression was one of the major reasons for emoji usage.

Past research also indicates that emojis can serve to clarify an online message and reduce ambiguities (Derks et al., 2008; Kaye et al., 2016; Tang and Hew, 2019). For example, in the study by Kaye et al. (2016), participants reported that another main reason for using emoticons (apart from emotion expression) was to disambiguate a message, especially when there was a possibility of ambiguity leading to negative consequences. In another study, Chen and Siu (2017) surveyed 347 young Chinese people and discovered that one major reason behind emoticon usage was to enhance communication accuracy and efficiency along with other reasons, such as emotional expression, sociability, and enjoyment. A recent study with non-face emojis has yielded similar findings (Riordan, 2017).

Apart from the reason of conveying emotion and clarifying messages, emojis and stickers can also be utilized to fulfill strategic purposes. For example, Japanese teenagers use emojis not only to express emotions but also to manipulate the communication climate and construct their aesthetic self (Sugiyama, 2015). Some teenagers selectively use emojis to avoid making others think they are cold or angry (using animal emojis such as cats to create a calm, cute, and soft ambience). Lee et al. (2016) confirmed that stickers, like emojis, are often used for expressing detailed information about emotions. Moreover, people also use emotions and stickers for strategic motives, such as self-representation and impression management (e.g., expressing oneself as one wishes to be seen by others, such as having a great sense of humor) (Derks et al., 2008; Lee et al., 2016).

While most research studies have focused solely on emojis or stickers, Zhou et al. (2017) conducted interviews and observations to investigate the combined use of emojis and stickers on WeChat, which is the most widely used instant messaging platform in the world (Lien and Cao, 2014). In addition to emotional expression and message clarification, WeChat users specified that they often send out emojis and stickers to avoid social awkwardness. Others also used emojis and stickers when they did not wish to talk (especially with older family members). In a more recent study, Tang and Hew (2019) conducted a comprehensive review of over 50 research studies and identified the main reasons for using emojis and stickers are to express emotions, clarify messages, and to fulfill other social purposes.

Just as people of different personality demonstrate vastly different behaviors when communicating face to face, past research also highlights that personality can play a role in determining online behaviors (Gosling et al., 2007; Amichai-Hamburger and Vinitzky, 2010; Eftekhar et al., 2014; Settanni and Marengo, 2015). For example, people high on agreeableness (willingness to cooperate and show warmth) are more concerned about self-presentation, such as being cooperative and friendly, during online interactions (Leary and Allen, 2011). People who score high on shyness (fear or worry about social interactions) showed higher preference for communicating online and more problematic internet use behaviors, such as surfing on the internet to avoid attending to stressful events (Ebeling-Witte et al., 2007). Other evidence also suggests that people’s personality judgments of users based on social media profiles are highly consistent with the users’ actual personality trait assessments (Gosling et al., 2007; Back et al., 2008; Wall et al., 2016).

As research findings converge to suggest that personality traits can affect human behaviors in CMC, there is also a line of research that specifically focuses on how personality can be portrayed through emoji and sticker usage patterns (Thompsen and Foulger, 1996; Kalyanaraman and Ivory, 2006; Kaye et al., 2017). Hall and Pennington (2013) discovered that the frequency of emoticon use in Facebook users is positively associated with extraversion (level of preference for social interactions) and self-monitoring traits. Marengo et al. (2017) found that the use of 36 out of 91 emojis is associated with three of the Big-five traits including neuroticism (level of emotional stability), extraversion, and agreeableness (willingness to cooperate and show warmth).

Li et al. (2018) further examined emoji usage patterns in 1.13 billion tweets for 352,245 users. It was found that people who are low on extraversion use emojis more often (as introverts prefer implicit visual contexts over explicit texts where they have to express themselves more directly). People who score higher on agreeableness and neuroticism and lower on conscientiousness (the tendency to self-discipline) also use emojis more often. In addition, people who are high on extraversion prefer positive emojis while those high on neuroticism prefer exaggerated and emotion rich emojis. Openness to experience (degree of appreciation for new experiences) had little correlation with emoji use patterns.

Together, past research findings indicate that people adopt emojis and stickers for different reasons, such as to express emotion, clarify messages, or to serve other strategic functions. It was also found that certain usage patterns for emojis are related to people’s personality traits. However, there were several questions that had remained unanswered in the current literature. Firstly, although personality traits had been found to be associated with emoji usage patterns, such as the selection and frequency of usage, no studies have examined if personality traits were related to people’s reasons for using emojis. Secondly, no research had examined if personality traits were associated with who people choose to use emojis and stickers with (i.e., peers or elderlies) and in what situation they tend to use emojis and stickers (i.e., private chat between two people or group chats that involves a number of individuals). Lastly, despite their increasing popularity, stickers did not receive the same empirical attention as emojis and very few studies had compared the differences in usage between these two. Since stickers are more complex in form and convey more complicated information, it is possible that they could be used differently from emojis (Tang and Hew, 2019).

In order to answer the remaining questions in the current literature, the present study aimed to explore (1) whether personality traits would be associated with people’s reasons for using emojis and stickers; (2) whether people who show different usage patterns (depending on the target and situation) would also differ in personality; and (3) whether emojis and stickers are used differently for people of different personality. In the present study, participants reported their reasons and patterns for using emojis and stickers in a questionnaire and completed personality tests that assessed their Big Five personality traits and shyness. The Big Five traits were assessed because past studies indicate they are related to emoji and sticker usage patterns or behaviors during CMC (Ebeling-Witte et al., 2007; Marengo et al., 2017; Li et al., 2018). Shyness was assessed separately because it is conceptually distinct from the dimensions of extraversion and neuroticism but has also been found to be related to people’s online behaviors (Briggs, 1988; Ebeling-Witte et al., 2007). It was hypothesized that (1) personality traits would be related to the reasons for using emojis and stickers; (2) people who show different usage patterns would differ in personality; and (3) emojis and stickers would be used differently for people of different personality.

## Materials and Methods

### Participants

A total number of 312 participants ranging from 16 to 58 years of age (*M*_age_ = 28.33, SD = 9.39) were recruited from a public advertisement released on WeChat. There were 102 males (33%) and 210 females (67%). Participants came from a range of backgrounds (22% high school students, 34% University students, 23% professional workers, and 21% non-professional workers).

Most participants were ethnically Chinese, and one was ethnically Korean. With regard to the geographical location of participants, 62% of participants were residing in China, and 38% were residing in other countries. For those who were living in China, the geographical distribution was Shanghai (64%), Yunnan (17%), Guangdong (6%), Guangxi (3%), Beijing (3%), Zhejiang (3%), Liaoning (2%), Hubei (1%), and Shanxi (1%). For participants who were living in other countries, the geographical distribution was the United Kingdom (52%), New Zealand (22%), the United States (16%), Japan (5%), France (3%), and Singapore (2%).

All participants spoke Mandarin, stated that WeChat was their most used instant messaging service, and indicated they had the habit of using emojis and stickers. The study only analyzed complete questionnaires so there were no missing cases. All participants gave informed consent to take part in the present study.

### Material and Procedure

All participants completed three anonymous questionnaires (combined into one online document under one rather than three separate titles) presented on a WeChat link. The first questionnaire was the “Emoji and Sticker Usage Pattern Questionnaire,” which contained 27 items. Participants first indicated their average frequency of usage for emojis and stickers on a 5-point scale (1 = below 5% in each conversation, 2 = 5–10% in each conversation, 3 = 10–15% in each conversation, 4 = 15–20% in each conversation, and 5 = above 20% in each conversation). This question asked participants to reflect on their last lengthy conversation or chatting style in general and report the percentages of messages (one message refers to one text bubble in WeChat) that contained at least one emoji. In this questionnaire, participants also reported the situation (i.e., in private or group chats) and with whom (i.e., friends, colleagues, family members, elderlies, and people in authority) they would be more or less likely to use emojis and stickers. Note that the above questions were first asked for emojis and then repeated for stickers.

Participants then completed the most important items that assessed their reasons for using emoji and stickers. Participants were required to rate on a 7-point scale (1 = not at all like me, 2 = not a lot like me, 3 = somewhat not like me, 4 = not sure, 5 = somewhat like me, 6 = a lot like me, and 7 = entirely like me) for each of the following reasons: I use emojis because (1) emojis can help me express emotion, (2) emojis can disambiguate and clarify messages, (3) emojis can lighten up the mood during a conversation, (4) emojis can show people that I am interesting and or/have a sense of humor, (5) emojis can help me avoid awkwardness and/or uncomfortable feelings in an online conversation, and (6) emojis can help me end a conversation when I have nothing more to say. Participants then had to choose one of the above reasons that they thought was the most prominent reason for using emojis (participants’ responses to this particular question was compared with their ratings in the previous questions and used to identify incongruent responses and participants who might give untruthful responses). These items were then repeated for stickers. This section of the questionnaire was important in assessing the key information about people’s reasons of usage. A 7-point-scale was adopted, as past research suggests that 7-point scales can measure participants’ evaluations more accurately, are more suited to electronic distribution of usability inventories, and can lead to higher reliability (Colman et al., 1997; Finstad, 2010). The main reasons for using emojis and stickers in the questionnaire were selected based on past research findings about why people use emojis and stickers, such as those by Kaye et al. (2016), Lee et al. (2016), Chen and Siu (2017), and Zhou et al. (2017). For all the items in this questionnaire, participants could add more information or explanation in a space provided under each item if they felt like the given options did not describe them well.

The second questionnaire was the Revised Cheek and Buss Shyness Scale (Cheek and Buss, 1981). It contained 13 items (translated to Mandarin Chinese) (Cronbach’s alpha coefficients: shyness α = 0.78). Each item included a statement (such as “I feel tense when I am with people I do not know well”), and participants rated how each statement described themselves on a 5-point scale (1 = very uncharacteristic or untrue, strongly disagree, 2 = uncharacteristic, 3 = neutral, 4 = characteristic, and 5 = very characteristic or true, strongly agree). In this questionnaire, items 3, 6, 9, and 12 were reversed so each participant’ responses were recoded before scoring. These reversed items were mainly used to identify incongruent response. Questionnaires with incongruent responses and overly similar responses (e.g., giving a rating of 1 for the majorly of items or all items) were regarded as incomplete, and they were not included in the final analyses. This shyness scale has been found to show sound psychometric properties across cultures (Crozier, 2005; Hopko et al., 2005).

The third questionnaire was the revised NEO-Five Factor Inventory (NEO-FFI; Costa and McCrae, 1992). Participants answered 60 questions (in Mandarin) assessing their Big Five personality traits of neuroticism, extraversion, openness to experience, agreeableness, and conscientiousness (Cronbach’s alpha coefficients: Neuroticism α = 0.85, Extraversion α = 0.82, Openness α = 0.65, Agreeableness α = 0.60, and Conscientiousness α = 0.83). In the questionnaire, participants rated each of the 60 statements (such as “I like talking to others” “I prefer working alone”) on a 5-point scale (1 = very unlike me, 2 = unlike me, 3 = not sure, 4 = like me, and 5 = like me very much). The questionnaire also contained 23 reversed items. The NEO-FFI has been adopted as a valid and reliable measure of personality (McCrae and Costa, 2004; Anisi, 2012) and has been found to be suitable for the Chinese population (McCrae and Costa, 1997; Yao and Liang, 2010).

## Results

### Preliminary Analyses

#### Frequency of Usage

For emojis, 17.3% of participants (*n* = 54) reported that on average, emojis occupied about 5% in each conversation, 18.9% (*n* = 59) used emojis between 5 and 10%, 19.5% (*n* = 61) used them between 10 and 15%, 23.1% (*n* = 72) used between 15 and 20%, and 21.2% (*n* = 66) used more than 20%. For stickers, 20.5% (*n* = 64) reported stickers took up to 5% in each conversation, 21.8% (*n* = 68) used stickers between 5 and 10%, 15.4% (*n* = 48) used between 10 and 15%, 23.1% (*n* = 72) used between 15 and 20%, and 19.2% (*n* = 60) used stickers for more than 20%. The frequency of usage for emojis was significantly correlated with frequency of usage for stickers, *r*_*s*_ = 0.74, *p* < 0.001.

#### The Most Prominent Reason of Usage

For emojis, 36.5% (*n* = 114) participants reported that they mainly used emojis to lighten up the mood in conversations, 27.6% (*n* = 86) participants used emojis to clarify messages, and 19.2% (*n* = 60) used emojis to express emotion. Apart from these three major reasons, 12.5% (*n* = 39) used emojis mainly to avoid awkwardness, while 4.2% (*n* = 13) used emojis mainly to end conversations. No participants indicated other reasons for using emojis in the questionnaire.

As for the most prominent reason for using stickers, 39.4% (*n* = 123) reported that they mainly used stickers to lighten up the mood in conversations, 24.0% (*n* = 75) mainly adopted stickers to clarify messages, and 15.1% (*n* = 47) used stickers mainly to avoid awkwardness. Thus, the first two main reasons for using stickers were similar to that of emojis, but the third main reason was different. In addition, 14.4% (*n* = 45) used stickers to express emotions while 7.1% (*n* = 22) used stickers to mainly end conversations. No participants indicated other reasons for using stickers in the questionnaire.

#### Target of Usage

For emojis, 73.1% (*n* = 228) participants indicated that they did not change their frequency of usage depending on who the target person was, and 26.9% (*n* = 84) reported that they would use emojis less for elderlies and people in authority. For stickers, 61.6% (*n* = 192) did not change frequency of usage for different targets and 38.4% (*n* = 120) reported that they used stickers less for elderlies and people in authority.

#### Situation of Usage

For emojis, 40.4% (*n* = 126) participants reported that they tend to use emojis more often in private chats, 7.7% (*n* = 24) tend to use emojis more in group chats and 51.9% (*n* = 162) did not discriminate between these two situations. For stickers, 53.8% (*n* = 168) participants reported a tendency to use them more often in private chats, 5.8% (*n* = 18) tend to use stickers in group chats, and 40.4% (*n* = 126) used stickers equally in private and group chats.

### Main Analyses

#### Personality Traits and Reasons for Using Emojis

The focal question of the present study was whether personality traits would be related to people’s reasons for using emojis and stickers. To explore possible associations between shyness and reasons for using emojis, bivariate correlations were conducted on shyness scores and the ratings participants assigned to each of the six main reasons. The analyses revealed a significant positive correlation between shyness and using emojis to avoid awkwardness (for detailed correlation results see Table 1).

**TABLE 1:** Bivariate correlation results showing the Spearman’s rank correlations (*r*_*s*_) between personality traits and reasons for using emojis.

	Express emotions	Clarify/disambiguate message	Lighten up mood	Show a sense of humor	Avoid awkwardness	End conversation
Shyness	0.06	–0.07	–0.10	–0.09	0.11*	–0.04
Neuroticism	0.07	0.10	0.03	–0.02	0.28**	0.11
Extraversion	–0.08	0.10	–0.01	0.09	−0.36**	–0.06
Agreeableness	0.31**	0.52**	0.42**	0.30**	–0.05	–0.06
Openness	–0.10	–0.003	–0.01	0.02	0.05	0.08
Conscientiousness	0.08	–0.07	–0.04	0.04	0.01	0.08

*p < 0.05, **p < 0.01.

Bivariate correlations were then conducted on each of the Big Five personality traits and reasons for using emojis. Results revealed that neuroticism was positively correlated with using emojis to avoid awkwardness, while extraversion was negatively correlated with using emojis to avoid awkwardness. Agreeableness was positively correlated with using emojis to express emotions, clarify messages, lighten up the mood, and show a sense of humor. There were no other significant correlations found for openness to experience and conscientiousness.

#### Personality Traits and Reasons for Using Stickers

Stickers were examined separately in the present study. Bivariate correlations showed that shyness was positively correlated with using stickers to avoid awkwardness and to end conversation. Shyness was also negatively correlated with using stickers to show a sense of humor (see Table 2). Participants’ neuroticism scores were positively correlated with using stickers to avoid awkwardness and to end conversation and negatively correlated with using stickers to show a sense of humor. Extraversion was positively correlated with using stickers to show a sense of humor and negatively correlated with using stickers to avoid awkwardness. Agreeableness was positively correlated with using stickers to express emotion, clarify messages, lighten up the mood and show a sense of humor. No other significant correlations were found for openness to experience and conscientiousness.

**TABLE 2:** Bivariate correlation results showing the Spearman’s rank correlations (*r*_*s*_) between personality traits and reasons for using stickers.

	Express emotions	Clarify/disambiguate message	Lighten up mood	Show a sense of humor	Avoid awkwardness	End conversation
Shyness	0.02	–0.01	0.08	−0.18**	0.27**	0.17**
Neuroticism	–0.08	–0.02	–0.02	−0.15*	0.27**	0.20*
Extraversion	0.05	0.02	–0.05	0.30**	−0.22**	–0.05
Agreeableness	0.41**	0.32**	0.21*	0.28**	0.04	–0.05
Openness	–0.07	–0.03	–0.04	–0.01	–0.03	0.02
Conscientiousness	0.04	–0.09	0.07	0.08	–0.01	0.07

*p < 0.05, **p < 0.01.

#### Personality Traits and Usage Patterns for Emojis and Stickers

**FIGURE 2:**
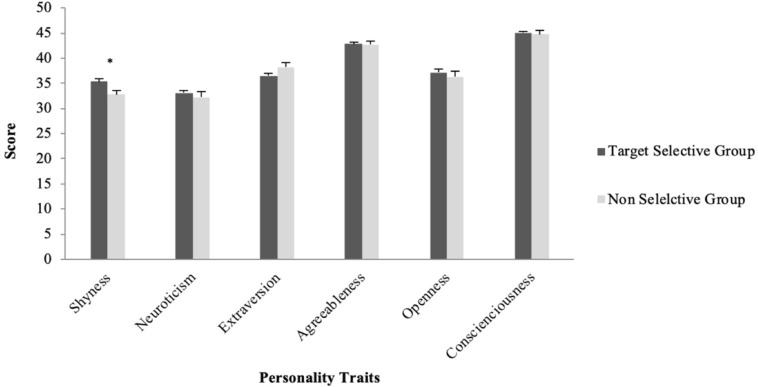
Average personality scores for participants who used emojis less with elderlies and people in authority (the Target Selective Group) and those who did not (the Non-Selective Group).

The second focal question of the present study was whether people of different personality would show different usage patterns. First, independent samples *t*-tests were performed to test if the shyness and the Big Five Personality trait scores would be different for participants who reported to use emojis less with elderlies and people in authority (the Target Selective Group) and those who did not report such a habit (the Non-Selective Group). Results showed the Target Selective Group had significantly higher shyness score (*M* = 35.34, SD = 7.69) than the Non-selective Group (*M* = 32.79, SD = 7.64) (*t*(310) = 2.61, *p* = 0.01, *d* = 0.33, *r* = 0.16). The two groups did not differ in the other personality traits (*p* > 0.05) (see Figure 2).

**FIGURE 3:**
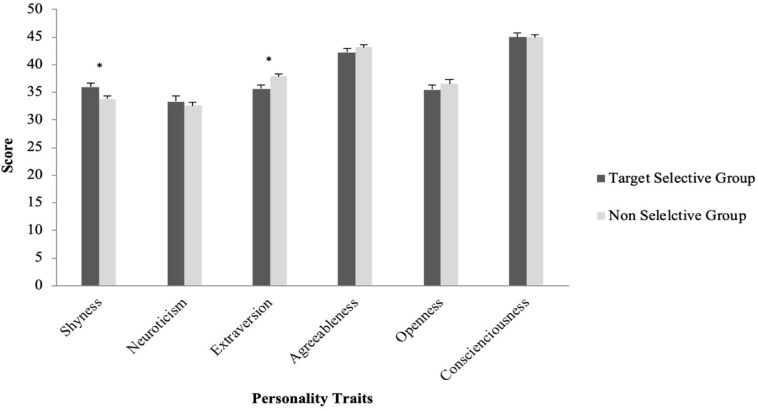
Average personality scores for participants who used stickers less with elderlies and people in authority (the Target Selective Group) and those who did not (the Non-Selective Group).

In terms of sticker usage, independent samples *t*-test results revealed that shyness score was significantly higher for the Target Selective Group (*M* = 35.95, SD = 8.43) than the Non-Selective Group (*M* = 33.84, SD = 7.20) (*t*(310) = 2.27, *p* = 0.02, *d* = 0.27, *r* = 0.13). The extraversion score was significantly higher for the Non-Selective Group (*M* = 37.88, SD = 6.83) than the Target Selective Group (*M* = 35.60, SD = 8.62) (*t*(310) = 2.45, *p* = 0.02, *d* = 0.29, *r* = 0.15). There were no significant differences in other Big Five personality traits between the two groups (*p* > 0.05) (see Figure 3).

**FIGURE 4:**
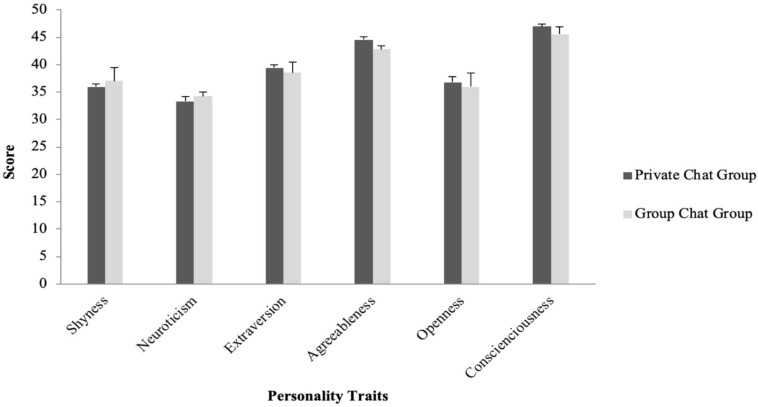
Average personality scores for participants who used emojis more in private chats (Private Chat Group) and more in group chats (Group Chat Group).

The present study also tested if people with different personality traits would use emojis and stickers more in private or in group chat situations. For emojis, an independent sample *t*-test was first conducted to compare whether personality scores would differ for participants who reported using emojis more in private chats (the Private Chat Group) and those who used emojis more in group chats (Group Chat Group). The *t*-test revealed no significant differences in personality traits between the two groups (*p* > 0.05) (see Figure 4).

**FIGURE 5:**
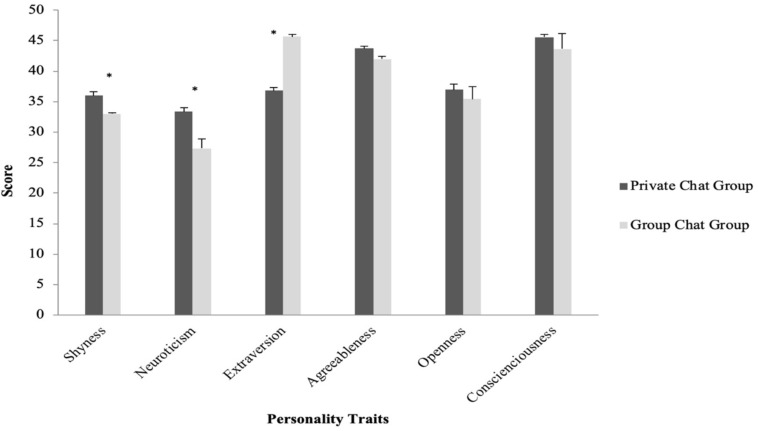
Average personality scores for participants who used stickers more in private chats (Private Chat Group) and more in group chats (Group Chat Group).

As for stickers, independent samples *t*-tests showed that the Private Chat Group had significantly higher shyness scores (*M* = 35.75, SD = 7.92) than those in the Group Chat Group (*M* = 33.00, SD = 8.84) (*t*(184) = 2.72, *p* = 0.01, *d* = 0.31, *r* = 0.15). The Private Chat Group also had significantly higher neuroticism scores (*M* = 33.32, SD = 8.82) than the Group Chat Group (*M* = 27.33, SD = 6.84) (*t*(184) = 3.42, *p* = 0.01, *d* = 0.76, *r* = 0.35). In contrast, The Group Chat Group had higher extraversion score (*M* = 45.67, SD = 1.28) than those in the Private Chat Group (*M* = 36.82, SD = 7.00) (*t*(184) = 14.29, *p* < 0.001, *d* = 0.77, *r* = 0.36) (see Figure 5).

## Discussion

The present study was designed to investigate whether personality traits were related to the reasons and patterns (with whom and in what situation) that people use emojis and stickers. Past research identified a number of reasons for the combined use of emojis and stickers (Tang and Hew, 2019). Other research also suggests that the Big Five personality traits, including extraversion, neuroticism, and agreeableness, are related to the frequency of emoji usage and people’s self-identification with common emojis (Hall and Pennington, 2013; Marengo et al., 2017; Li et al., 2018). The present study examined people’s reasons for using emojis and stickers separately and expands the current literature by revealing that personality traits are associated with different reasons for using emojis and stickers. In addition to the Big Five personality traits, the present study also closely examined shyness for the first time. The key results showed that people who score higher in shyness and neuroticism tend to be more likely to use emojis to avoid awkwardness in a conversation. In contrast, people high on extraversion are less likely to use emojis to avoid awkwardness.

Similar to what has been found with emojis, people who are shyer and more neurotic are more likely to use stickers to avoid awkwardness. However, these individuals are also less likely to use stickers to show others that they are interesting or have a great sense of humor. It is also important to note that shyness and neuroticism were both positively associated with the reason of using stickers to end a conversation. This was only observed for stickers but not for emojis. Therefore, people who are shyer and more neurotic have a higher tendency to use stickers to end a conversation when they run out of words to say. Moreover, people who are high on extraversion are less likely to use stickers to avoid awkwardness, and this association was similar to emojis. However, people with higher level of extraversion are also more likely to use stickers to show their sense of humor. This was also not observed with emojis. Thus, the present study further reveals that emojis and stickers can be adopted for different reasons by people who differ in personality.

People who are extraverts are more gregarious and enjoy social interactions (Eysenck, 1973). Thus, for these individuals, emojis and stickers could be adopted as tools to express themselves (such as showing others they have a sense of humor). Different to extraverts, people who are shy are more likely to experience apprehension and awkwardness or lacking comfort when around other individuals (Heiser et al., 2009). People who are neurotic are more likely to experience negative emotions, such as anxiety, worry, and frustration, and tend to perceive ordinary situations as threatening (Lahey, 2009). These individuals may be more likely to feel uncomfortable in online conversations and thus use emojis and stickers to avoid awkwardness and end conversations. The higher tendency of these people to use stickers to end conversations could be because stickers are always sent separately without any texts (suitable when these individuals run out of words to say) and are often more complex in form (Lee et al., 2016; Zhou et al., 2017). For example, a goodbye sticker can be presented as a person saying goodbye while crying or saying that “I am busy studying,” thus providing more explanatory information than just saying goodbye (see Figure 1 for examples). These features might render stickers more appropriate for ending a conversation compared to emojis that convey relatively simple information (Cramer et al., 2016).

With regard to the other personality traits, people who are high on agreeableness are more likely to use emojis and stickers to express emotions, clarify messages, lighten up the mood, and show a sense of humor. High agreeableness is characterized by warmth, tendermindedness and high level of cooperation (Graziano and Eisenberg, 1997). People who possess these characteristics could be more concerned about using emojis and stickers to enhance the conversation (i.e., clarify a message and lightening up the mood) or to express themselves to others (i.e., representing their emotions and showing humor). It is worth mentioning that openness to experiences and conscientiousness were not associated with any reasons of usage. This finding was consistent with past studies that also failed to obtain major associations for these two personality traits (Li et al., 2018). This could be because openness to experience and conscientiousness are not related to emotional expression and processing but instead have strong association with people’s general cognitive ability (Côté and Moskowitz, 1998; LePine et al., 2000; Elliot and Thrash, 2002; Moutafi et al., 2006; Robinson, 2007).

Past research also indicates that people tend to use emojis and stickers in different context (e.g., people tend to use positive emojis in asynchronous communication and negative emojis in synchronous contexts) (Tang and Hew, 2019). The present study further suggests that that some people also adjust usage patterns depending the size of the chat group and who the target person is. Specifically, the present findings suggest that people who selectively use emojis more in private or group chats do not show any significant personality differences. However, people who choose to use stickers more frequently in private chats are shown to be shyer and more neurotic. In contrast, those who are more likely to use stickers in group chats have higher extraversion scores. Moreover, some participants also reported that they would reduce usage of emojis and stickers for elderlies and people in authority. People who had such a habit have higher shyness scores and lower extraversion scores. Together, these results reflect personality traits are related to different usage patterns for emojis and stickers.

Understanding how people with different personality traits use emojis and stickers can have real-world implications. Lee et al. (2008) used emoji-based instruments to detect signs of depression in stroke patients and found these items can provide a reliable measure of depressive symptoms. Marengo et al. (2017) suggest that new forms of communication, such as emojis, could have the potential to at least partly replace traditional assessment tools of individual personality differences. Thus, the associations found in the present study could provide valuable information about how individual differences can be reflected by why and how people use emojis. This could in turn aid the development of emoji-based or new types of personality assessments. In addition, as stickers have become increasingly popular, it is also important to look into how they unveil personality and whether their usage patterns differ from traditional emojis. This would promote better understanding about how these “new generation emojis” serve to facilitate human communication.

The present study has several limitations. First, although the online questionnaire was presented as one document, it actually consisted of three separate questionnaires which were of considerable length. Many participants found the questionnaire time consuming thus the study only had a relatively small sample. Second, male and female participants were not in the same proportion. As gender was found to influence the pattern of emoticon use (Wolf, 2000; Tossell et al., 2012), it is important that future studies try to include an equal number of males and females. Third, the present study relied on self-report data thus was subject to the effect of untruthful responses and social desirability. Existing studies have used simulated real-life communications (e.g., email exchanges) to test the effect of emojis on sender impression formation (Byron and Baldridge, 2007). Thus, future studies that investigate emoji and sticker usage patterns could also involve participants in a more real CMC context (e.g., making participants chat online with another person and observe how they use emojis and stickers). Fourth, the sample only consisted of participants who were ethnically Chinese. As culture has been found to determine online behavior to some extent (Kralisch and Berendt, 2004), it is important that future studies examine participants from different cultures and examine how culture could influence how people use emojis and stickers. Last, there are a number of questions that cannot be answered by findings in the present study. Future studies can (1) further investigate the correlations between usage patterns of emojis and stickers, especially within the same person, (2) test what functions stickers and emojis can serve when they are used at the same time, (3) explore people’s choices between emojis and stickers in CMC and the rationale behind their preferences, and (4) examine the use of sticker more closely to see if they would generate any unique speech act functions that were not identified in the present study.

In summary, the present study highlights that personality traits including shyness, neuroticism, extraversion, and agreeableness are associated with different reasons of using emojis and stickers. People who differ in these traits also tend to change their frequency of usage depending on the target person and the situation. In addition, emojis and sticker usage patterns are generally associated with personality traits in similar ways, but some differences do exist. These key findings provide more detailed evidence about how emojis and stickers can help people with different personality traits to interact and achieve different communicative purposes online.

## Data Availability Statement

The datasets generated for this study are available on request to the corresponding author.

## Ethics Statement

The studies involving human participants were reviewed and approved by Shanghai International Studies University. The participants provided their written informed consent to participate in this study.

## Author Contributions

SL developed the study concept and created the study design, performed the data analysis and interpretation, and drafted the manuscript. Both authors conducted the data collection and approved the final version of the manuscript for submission.

## Conflict of Interest

The authors declare that the research was conducted in the absence of any commercial or financial relationships that could be construed as a potential conflict of interest.
